# Photosynthesis and Photosynthetic Electron Flow in the Alpine Evergreen Species *Quercus guyavifolia* in Winter

**DOI:** 10.3389/fpls.2016.01511

**Published:** 2016-10-20

**Authors:** Wei Huang, Hong Hu, Shi-Bao Zhang

**Affiliations:** Key Laboratory of Economic Plants and Biotechnology, Kunming Institute of Botany, Chinese Academy of SciencesKunming, China

**Keywords:** alpine evergreen broadleaf species, alternative electron flow, low night temperature, mesophyll conductance, photorespiration, photosynthesis

## Abstract

Alpine evergreen broadleaf tree species must regularly cope with low night temperatures in winter. However, the effects of low night temperatures on photosynthesis in alpine evergreen broadleaf tree species are unclear. We measured the diurnal photosynthetic parameters before and after cold snap for leaves of *Quercus guyavifolia* growing in its native habitat at 3290 m. On 11 and 12 December 2013 (before cold snap), stomatal and mesophyll conductances (*g*_s_ and *g*_m_), CO_2_ assimilation rate (*A*_n_), and total electron flow through PSII (*J*_PSII_) at daytime were maintained at high levels. The major action of alternative electron flow was to provide extra ATP for primary metabolisms. On 20 December 2013 (after cold snap), the diurnal values of *g*_s_, *g*_m_, *A*_n_, and *J*_PSII_ at daytime largely decreased, mainly due to the large decrease in night air temperature. Meanwhile, the ratio of photorespiration and alternative electron flow to *J*_PSII_ largely increased on 20 December. Furthermore, the high levels of alternative electron flow were accompanied with low rates of extra ATP production. A quantitative limitation analysis reveals that the *g*_m_ limitation increased on 20 December with decreased night air temperature. Therefore, the night air temperature was an important determinant of stomatal/mesophyll conductance and photosynthesis. When photosynthesis is inhibited following freezing night temperatures, photorespiration and alternative electron flow are important electron sinks, which support the role of photorespiration and alternative electron flow in photoportection for alpine plants under low temperatures.

## Introduction

Plants growing at high elevations must cope with extreme weather conditions in winter, such as high light intensity combined with low temperature (chilling or freezing). In alpine regions, low temperature is a primary determinant of species distribution and growth. Physiological and metabolic processes decrease at temperatures lower than the optimum (Sage et al., 2008). Low temperature depresses the rate of photosynthesis both directly and indirectly, either by influencing the activity of photosynthetic enzymes involved in the Calvin cycle, such as Rubisco (Yamori et al., 2010), or by reducing stomatal conductance (Allen et al., 2002). In cold-tolerant species, the photosynthetic rate at low temperature is tended to be limited by RuBP carboxylation (Yamori et al., 2010). During winter in alpine zones, low temperatures during the daytime likely restrict photosynthesis in evergreen broadleaf tree species.

Low night temperature is another temperature stress for alpine evergreen broadleaf tree species in winter, and its effect on photosynthesis has been examined in several tropical and subtropical species, e.g., tomato (*Lycopersicon esculentum*; Martin et al., 1981), coffee (*Coffea arabica*; Bauer et al., 1985), grape (*Vitis vinifera*; Flexas et al., 1999), and mango (*Mangifera indica*; Allen et al., 2002; Elsheery et al., 2007, 2008). Usually, stomatal conductance and net photosynthesis are inhibited during the day following an overnight temperature of 5 to 7°C. For cold-tolerant conifer Engelmann spruce (*Picea engelmannii* Parry), exposure to −2.5°C in the dark for 10 h causes a slight but reversible reduction in gas-exchange parameters during the ensuing days (Delucia, 1987). Alpine evergreen broadleaf species usually experience night chilling temperatures in winter. However, the effect that this stress has on their stomatal conductance and photosynthesis needs further study. In addition, subzero night air temperatures are another severe temperature stress in winter for alpine evergreen tree species. Because subzero night temperatures in alpine zones can reduce leaf and stem hydraulic conductance, researchers have speculated that these freezing conditions can further limit stomatal conductance and, thus, suppress photosynthesis. Therefore, study about how freezing night temperatures influence stomatal conductance, and photosynthesis in alpine evergreen broadleaf tree species is on time.

Mesophyll conductance is another important determinant of photosynthesis in plants (Carriquí et al., 2015), especially under environmental stresses (Flexas et al., 2002; Scafaro et al., 2011). During periods of drought, the decline in photosynthetic rates in grape is partially due to decreased mesophyll conductance (Flexas et al., 2002). At low temperatures, reduced levels of mesophyll conductance limit photosynthetic CO_2_ assimilation in tobacco (*Nicotiana tabacum*; Walker et al., 2013) and *Oryza* species (Scafaro et al., 2011). Although, the temperature response of mesophyll conductance has been widely examined in tobacco and *Arabidopsis thaliana*, the temperature response of mesophyll conductance in alpine tree species is not known. Furthermore, previous studies on temperature responses of mesophyll conductance focus on leaf temperatures during daytime, but the impact of night temperatures on mesophyll conductance is little known. Alpine evergreen broadleaf species usually endure freezing or chilling night temperatures in winter, but it is unclear whether this temperature stresses induce a decline in their mesophyll conductance.

Plants growing at high elevations have several mechanisms for photoprotection, such as photorespiration (Streb et al., 2005), cyclic electron flow (Huang et al., 2015a), alternative electron flow (Streb et al., 2005; Laureau et al., 2013), and antioxidant systems (Streb et al., 2003). In a widely studied alpine herb, *Ranunculus glacialis*, excess electrons are transferred to oxygen when photorespiration is blocked, due to a high content of plastid terminal oxidase (PTOX) (Laureau et al., 2013). This PTOX has the capacity to transfer electrons from plastoquinone directly to oxygen and, thus, avoid a reduction in the plastoquinone pool, thereby protecting the chloroplasts from over-reduction (Joët et al., 2002; Josse et al., 2003; Laureau et al., 2013). Furthermore, the PTOX content is largely decreased during de-acclimation by *R. glacialis* plants, suggesting an important role for alternative electron sinks in acclimating to their habitat in alpine plants (Streb et al., 2005). Most previous studies about the photosynthetic regulation of alpine plants focus on deciduous herbs, such as *R. glacialis*. However, little is known about how photosynthetic electron flow is regulated in alpine evergreen broadleaf tree species during the winter, especially under condition of freezing night temperatures.

*Quercus guyavifolia* is one of the major alpine broadleaf tree species that native to high altitudes in southwest China. The optimum leaf temperature for photosynthesis of *Q. guyavifolia* decreased with increasing growth altitude, suggesting that *Q. guyavifolia* showed significantly plasticity of optimum leaf temperature for photosynthesis (Zhang et al., 2007). The optimum leaf temperature for photosynthesis of *Q. guyavifolia* was about 20°C in summer when grown at an altitude of 3180 m (Zhang et al., 2007). To determine the winter photosynthetic characteristics of the alpine tree species *Q. guyavifolia*, we evaluated gas exchange and chlorophyll fluorescence on 3 days that differed in air temperatures. The following questions were addressed: (1) Does a low night temperature affect stomatal and mesophyll conductance in this species; (2) How does *Q. guyavifolia* regulate photosynthetic electron flow and optimize photosynthesis in winter?

## Materials and methods

### Site and plant material

This study was conducted at the Lijiang Forest Ecosystem Research Station, Yunnan, China (3290 m, 27°00′11″N, 100°10′49″E). *Q. guyavifolia* H. Léveillé is an endemic tree species that grows in mountainous oak forests or pine-oak mixed forests of southwestern China at elevations of 2500–4300 m. As an important component of evergreen broad-leaved forests, plants of this species can reach 15 m height. For our investigation, we sampled 3- to 4-m-tall trees. At least five independent plants were used for photosynthetic measurements. The diurnal experiments were conducted at the end of 2013 (December 2013). The ambient temperature was recorded by an automatic meteorological station. According to the continuous records in 2013 of Lijiang Forest Ecosystem Research Station, the mean annual air temperature is 9.22°C and the annual precipitation is 1213 mm.

### Gas exchange and chlorophyll fluorescence measurements

Parameters for gas exchange and chlorophyll fluorescence were monitored with an open gas exchange system that incorporated infrared CO_2_ and water vapor analyzers (Li-6400XT; Li-Cor Biosciences, Lincoln, NE, USA) and a 2-cm^2^ measuring head (6400-40 Leaf Chamber Fluorometer; Li-Cor Biosciences). The atmospheric CO_2_ concentration was maintained at 400 μmol mol^−1^ by the Li-6400XT. To compare with the seasonal change in photosynthetic rate, light-saturated rate of photosynthesis and the light-saturation point of *Q. guyavifolia* in summer should be know. As a result, light response curves were measured in the morning (9:00 to 11:00 a.m.) in August 2015 (summer). After light adaptation at 1000 μmol photons m^−2^ s^−1^ for at least 20 min, light-adapted photosynthetic parameters were recorded after 2 min exposure to each light intensity (2000, 1600, 1200, 1000, 800, 600, 400, 300, 200, 100, 50, 20, and 0 μmol photons m^−2^ s^−1^).

The diurnal responses of net CO_2_ assimilation (*A*_n_) and stomatal conductance (*g*_s_) were determined at 2-h intervals between 07:00 h (dawn) and 17:00 h (dusk). To avoid any confounding due to leaf age, only mature leaves that had emerged in spring of 2013 were sampled. Before measurements began, the ambient photosynthetic photon flux density (PPFD) to which the leaves were exposed was determined by a micro-quantum sensor connected to the Li-6400XT. Actinic LED light (Li-6400XT) corresponding to the natural PPFD at a given solar time was used. The water vapor concentration in the measuring head was consistent with the ambient air humidity. Leaf temperature, relative air humidity, and vapor pressure deficit (VPD) were recorded automatically by the Li-6400XT. Each individual measurement was accomplished within 1 min.

Concurrent with our gas exchange measurements, the diurnal response of chlorophyll fluorescence was also checked. Fluorescence parameters *F*_o_′, *F*_m_′, and *F*_*s*_ were evaluated as previously described (Baker and Rosenqvist, 2004), with *F*_o_′ and *F*_m_′ representing the minimum and maximum fluorescence, respectively, after light-adaption, and *F*_*s*_ being the light-adapted steady-state fluorescence. The following calculations were used: maximum quantum yield of PSII after light adaptation, *F*_v_′/*F*_m_′ = (*F*_m_′ − *F*_o_′)/*F*_m_′; coefficient of PSII photochemical quenching, qP = (*F*_m_′ − *F*_*s*_)/(*F*_m_′ − *F*_o_′); effective quantum yield of PSII, Φ_PSII_ = (*F*_m_′ − *F*_*s*_)/*F*_m_′ (Genty et al., 1989).

### Estimation of photosynthetic electron flow

The electron transport using the data obtained by chlorophyll fluorescence measurements (*J*_PSII_) was computed as (Krall and Edwards, 1992):

$$J_{\text{PSII}}\;=\text{ PPFD }\times \;\Phi_{\text{PSII}}\times \;d\text{II }\times \text{ p}$$

where PPFD represents the incident photosynthetic photons flux density, Φ_PSII_ represents the effective quantum yield of PSII, *d*II represents the distribution ratio of light absorbed by chloroplast to PSII and is assumed to be 0.5, p represents the ratio of light absorbed by chloroplasts and is assumed to be 0.85.

The electron flow devoted to RuBP oxygenation (*J*_O_) or RuBP carboxylation (*J*_C_) was calculated according to the method of Valentini et al. (1995):

$$\begin{array}{l} \;J_{\text{C}}\;=\;1/3\;\times \;(J_{\text{PSII}}\;+8\;\times \;(A_{\text{n}}\;+\;R_{\text{d}}))\\ J_{\text{O}}\;=\;2/3\;\times \;(J_{\text{PSII}}\;-4\;\times \;(A_{\text{n}}\;+\;R_{\text{d}})) \end{array}$$

where *A*_n_ represents the measured CO_2_ assimilation rate, *R*_d_ represents the rate of mitochondrial respiration and was measured after 5 min dark adaptation.

The NADPH demands from CO_2_ assimilation and photorespiration were calculated according to the models of Farquhar et al. (1980). From the data from gas exchange measurements, the rate of electron transport for NADPH required by carboxylation and oxygenation of RuBP (*J*_g_) was calculated as follows (Zivcak et al., 2013; Walker et al., 2014):

$$J_{\text{g}}\;=\text{ 4 }\times \;\left(A_{\text{n}}\;+\;R_{\text{d}}\right)\;\times \;\left(C_{\text{i}}\text{ +2}\Gamma^{*}\right)\text{/}(C_{\text{i}}\;-\;\Gamma^{*})$$

where *C*_i_ is the intercellular CO_2_ concentration, Γ^*^ is the CO_2_ compensation point in the absence of day respiration, and be calculated according to the method of Long and Bernacchi (2003).

The alternative electron flow devoted to the water-water cycle (WWC) was calculated as follows (Makino et al., 2002; Zivcak et al., 2013):

$$\;J_{\text{a}}=J_{\text{PSII}}\;-\;J_{\text{g}}$$

### Estimations of mesophyll conductance and chloroplast CO_2_ concentration

We compared mesophyll conductance (*g*_m_) on three dates when PPFD was higher than 1000 μmol photons m^−2^ s^−1^. Values for *g*_m_ were estimated through a combination analysis of gas exchange and Chl fluorescence, according to the following equation (Harley et al., 1992; Loreto et al., 1992; Warren and Dreyer, 2006; Yamori et al., 2010):

$$g_{\text{m}}=\frac{A_{\text{n}}}{C_{\text{i}}\;-\Gamma^{*}\;(J_{\text{T}}\;+8(A_{\text{n}}\;+\;R_{\text{d}}))/(J_{\text{T}}\;-4(A_{\text{n}}\;+\;R_{\text{d}}))}$$

where *C*_i_ was the intercellular CO_2_ concentration, Γ^*^ was the CO_2_ compensation point in the absence of daytime respiration (Farquhar et al., 1980; Brooks and Farquhar, 1985) and estimated according to the method of Long and Bernacchi (2003). Using the estimated *g*_m_, we calculated the chloroplast CO_2_ concentration (*C*_c_) with the following equation (Long and Bernacchi, 2003; Warren and Dreyer, 2006; Yamori et al., 2010):

$$C_{\text{c}}=C_{\text{i}}\;-\;\frac{A_{\text{n}}}{g_{\text{m}}}$$

### Quantitative limitation analysis of *A*_n_

Photosynthetic limitations in *Q. guyavifolia* were assessed according to the method of Grassi and Magnani (2005) and Carriquí et al. (2015), which requires knowledge of *A*_n_, *g*_s_, *g*_m_, and *C*_c_. Using this method, relative photosynthetic limitations of stomatal (*l*_s_), mesophyll conductance (*l*_mc_) and biochemical (*l*_b_) are calculated, which represent a measure of the relative importance of stomatal diffusion, mesophyll diffusion and photosynthetic biochemistry in setting the observed value of *A*_n_. Relative photosynthetic limitations were calculated as follows (Grassi and Magnani, 2005; Carriquí et al., 2015):

$$\begin{array}{l} l_{\text{s}}=\frac{g_{\text{tot}}/g_{\text{s}}\;\times \;\partial A_{\text{n}}/\partial C_{\text{c}}}{g_{\text{tot}}\;+\;\partial A_{\text{n}}/\partial C_{\text{c}}}\\ l_{\text{mc}}=\frac{g_{\text{tot}}/g_{\text{m}}\;\times \;\partial A_{\text{n}}/\partial C_{\text{c}}}{g_{\text{tot}}\;+\;\partial A_{\text{n}}/\partial C_{\text{c}}}\\ l_{\text{b}}=\frac{g_{\text{tot}}}{g_{\text{tot}}\;+\;\partial A_{\text{n}}/\partial C_{\text{c}}} \end{array}$$

where *g*_tot_ is total conductance to CO_2_ between the leaf surface and carboxylation sites and calculated as: 1/*g*_tot_ = 1/*g*_s_ + 1/*g*_m_ (Grassi and Magnani, 2005). Because *A*_n_ reached the maximum values at 11:00 on 11, 12, and 20 December, we used the photosynthetic parameter at 11:00 to analyze the relative photosynthetic limitations on these 3 days.

### Modeling ATP supplied via flexible mechanisms

The total amount of ATP demand from Rubisco carboxylation and oxygenation was obtained with the following formula (Walker et al., 2014):

$$v_{\text{ATP}}=\frac{\left(A_{\text{net}}\;+\;R_{\text{d}})\;(3C_{\text{i}}\;+\;3.5\frac{\Gamma^{*}}{\alpha }\right)}{\left(C_{\text{i}}\;-\;\Gamma^{*}\right)}$$

where α is the ratio of CO_2_ release per *v*_o_, typically assumed to be 0.5.

Assuming that the stoichiometry of ATP/NADPH produced by LEF (electron transport from PSII to NADP^+^) is 1.29 (Sacksteder et al., 2000; Seelert et al., 2000; Walker et al., 2014), the amount of ATP produced by LEF was calculated as follows (Huang et al., 2016):

$$\;v_{\text{ATP}(\text{LEF})}=\;1/2\;\times \;J_{g}\;\times \;1.29$$

Rates of ATP supply from other flexible mechanisms such as cyclic electron flow and the WWC were determined by subtracting the amount of ATP produced by LEF from *v*_ATP_ according to Huang et al. (2016):

$$\;v_{\text{ATP}(\text{Flex})}=v_{\text{ATP}}\;-\;v_{\text{ATP}(\text{LEF})}$$

### Statistical analysis

The mean ± SE was calculated from five plants. We used one-way ANOVA and SPSS 16.0 software (SPSS Inc., Chicago, IL, USA) to examine differences among measurements made on 11, 12, and 20 December 2013, with those differences being considered significant at *P* < 0.05.

## Results

**Figure 1:**
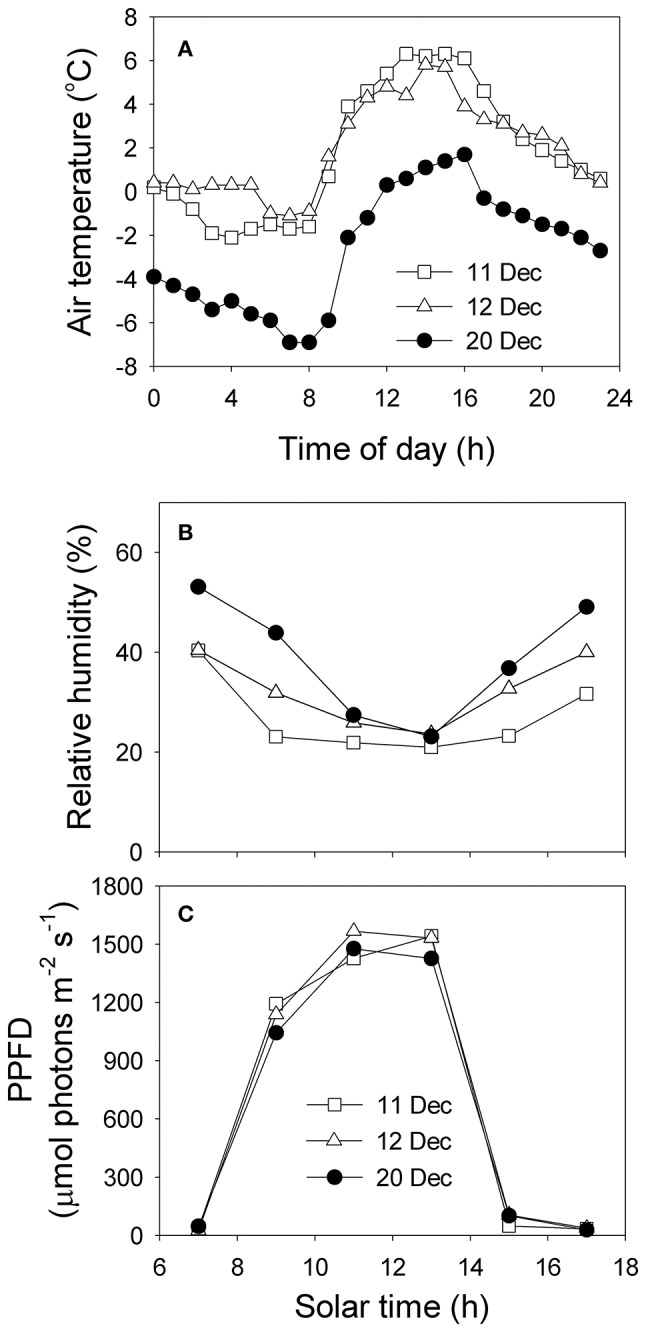
**Diel air temperature (A) and diurnal variations in relative air humidity (B) and photosynthetic photon flux density (PPFD) (C) on 11, 12, and 20 December 2013**.

**Figure 2:**
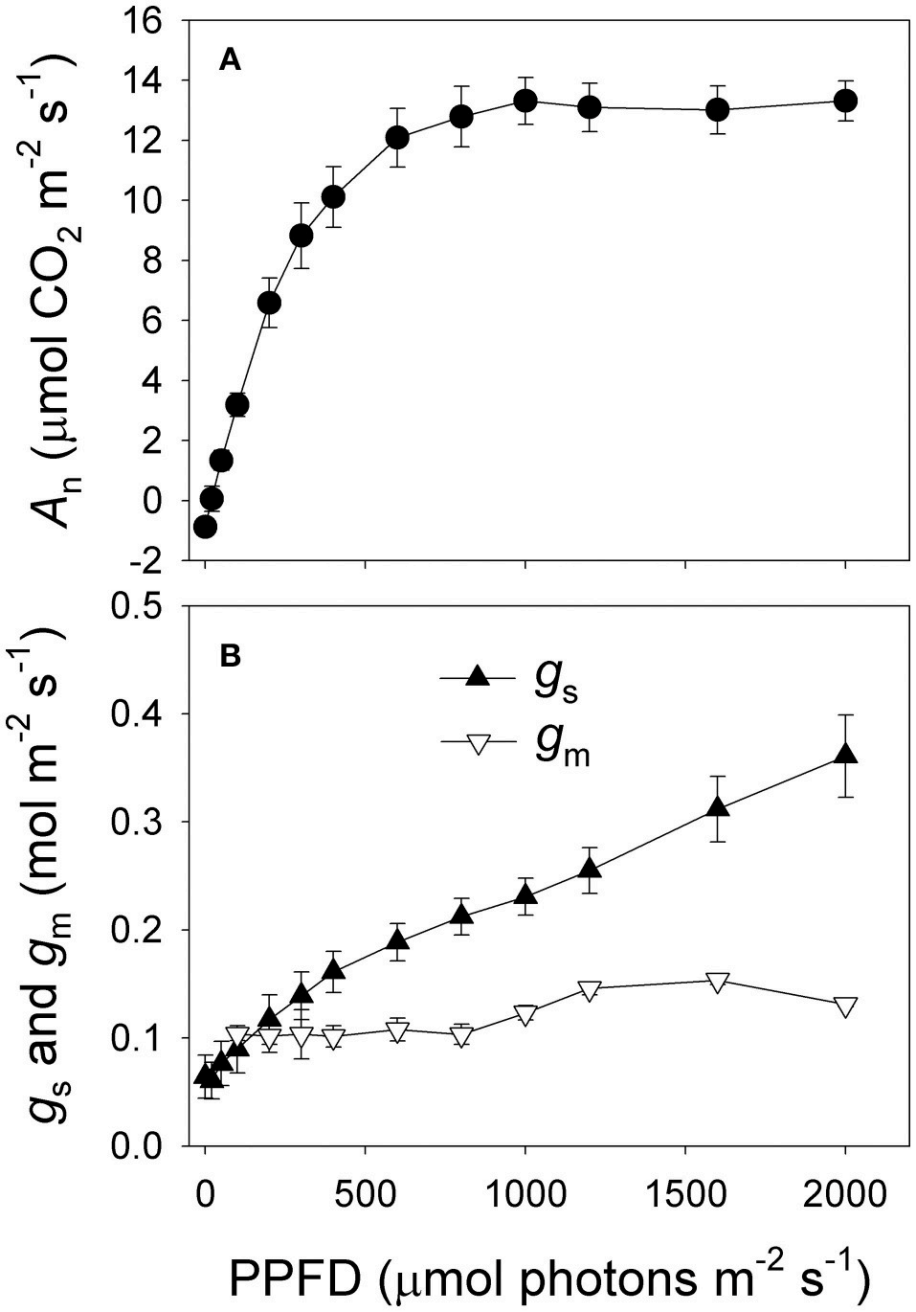
**Light response changes in net CO_2_ assimilation rate (*A*_n_) (A), stomatal conductance (*g*_s_) and mesophyll conductance (*g*_m_) (B) measured in summer (August 2015)**. Values are means ± SE (*n* = 5).

Trees of *Q. guyavifolia* experienced extreme micro-meteorological conditions in their native habitat over the diel period. On 11 and 12 December, the minimum air temperature at night was approximately −1°C while the maximum daytime air temperature was approximately 6°C (Figure 1). However, on 20 December, after a snowfall, the temperature decreased rapidly to approximately −7°C at night due to the cold snap, followed by a maximum daytime temperature of approximately 2°C (Figure 1). Relative humidity was 20 to 30% at midday during that winter season (Figure 1B). Within 2 h after dawn, PPFD increased from zero to >1000 μmol photons m^−2^ s^−1^, and the strongest PPFD was approximately 1500 μmol photons m^−2^ s^−1^ (Figure 1C). This high level of irradiance was maintained until 14:30 h. Light response curves indicated that the maximum rate of photosynthesis in *Q. guyavifolia* leaves occurs at approximately 1000 μmol photons m^−2^ s^−1^ (Figure 2A). The light-saturated rate of photosynthesis in summer was approximately 13 μmol m^−2^ s^−1^. In summer, stomatal conductance gradually increased with increasing light intensity and mesophyll conductance was higher than 0.1 mol m^−2^ s^−1^ (Figure 2B).

**Figure 3:**
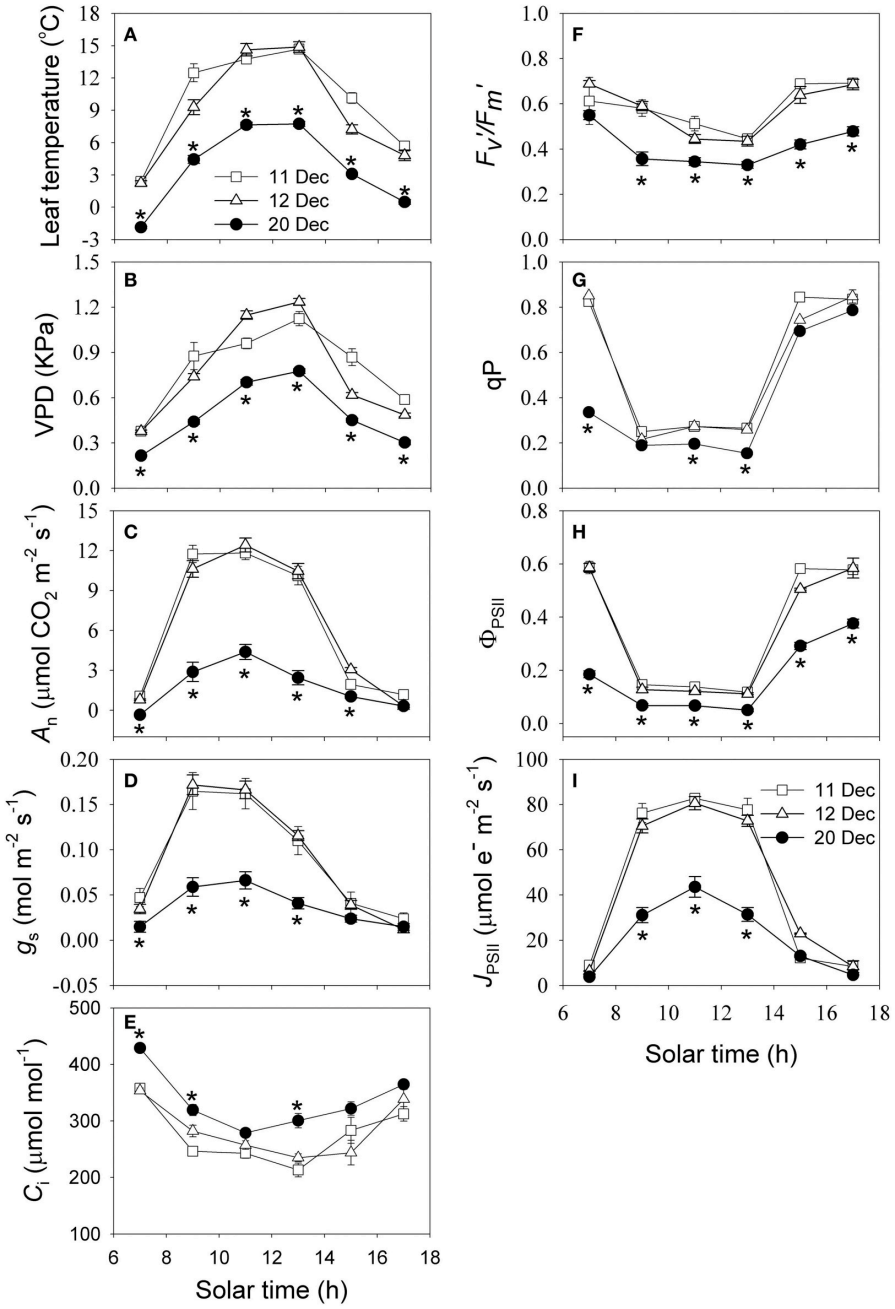
**Diurnal measurements of (A) leaf temperature, (B) vapor pressure deficit (VPD), (C) net CO_2_ assimilation rate (*A*_n_), (D) stomatal conductance (*g*_s_), (E) intercellular CO_2_ concentration (*C*_i_), (F) maximum quantum yield of photosystem II after light adaptation (*F*_v_′/*F*_m_′), (G) coefficient of photochemical quenching (qP), (H) effective quantum yield of photosystem II (Φ_PSII_), (I) electron flow through PSII calculated using data of chlorophyll fluorescence (*J*_PSII_) on 11, 12, and 20 December 2013**. Values are means ± SE (*n* = 5). Asterisks indicate significant differences on 20 December when compared to 11 and 12 December.

From dawn to 11:00 h, leaf temperature followed a trend similar to PAR, peaking at 11:00 h (Figure 3A). On 11 and 12 December, the maximum leaf temperature was approximately 15°C, but was only 8°C a few days later, on 20 December. Leaf temperature reached the maximum level during the period from 11:00 to 13:00. The vapor pressure deficit (VPD) rapidly increased from dawn to noon before peaking at 13:00 h (Figure 3B). In winter, the maximum VPD value was typically less than 1.2 kPa. After snow fell, this decline in leaf temperature and rise in relative humidity led to a decrease in VPD. Photosynthetic CO_2_ assimilation (*A*_n_) increased rapidly as PPFD strengthened from dawn to 11:00 h, reaching the maximum values of 12.4 and 11.8 μmol m^−2^ s^−1^ on 11 and 12 December, respectively (Figure 3C). On these two days, *A*_n_ was higher than 10 μmol m^−2^ s^−1^ during the period from 9:00 to 13:00. Following the snowfall, *A*_n_ declined significantly and reached a maximum of 4.4 μmol m^−2^ s^−1^ on 20 December (Figure 3C). The diurnal change in stomatal conductance (*g*_s_) exhibited a trend similar to *A*_n_, peaking at 0.17, 0.16, and 0.07 mol m^−2^ s^−1^ on 11, 12, and 20 December, respectively (Figure 3D). Values for both *g*_s_ and *A*_n_ were significantly lower on 20 December than on the previous two dates. Due to the higher rate of photosynthesis on 11 and 12 December, the diurnal intercellular CO_2_ concentration (*C*_i_) was lower on these 2 days than 20 December (Figure 3E).

During clear days, the light-adapted maximum quantum yield of PSII (*F*_*v*_′/*F*_*m*_′) decreased with increasing PAR (Figure 3F). On 11 and 12 December, diurnal values for *F*_*v*_′/*F*_*m*_′ were significantly higher than on 20 December. Because *F*_*v*_′/*F*_*m*_′ is inversely related to non-photochemical quenching (NPQ), this result seems to suggest NPQ was highly activated when CO_2_ was strongly restricted on the third date. Similar to the trend for *F*_*v*_′/*F*_*m*_′, qP was significantly decreased on 20 December (Figure 3G), indicating that the ability of the leaves to utilize the products of linear electron transport was significantly restricted on 20 December. Because effective quantum yield of PSII photochemistry (Φ_PSII_) is the product of *F*_*v*_′/*F*_*m*_′ and qP, the decreases in both of those variables led to a decrease in Φ_PSII_ in leaves on the third date (Figure 3H). The diurnal change in electron transfer rate through PSII (*J*_PSII_) followed a trend similar to *A*_n_, peaking at 11:00 h. Maximum *J*_PSII_ values were 80.6, 85.2, and 43.5 μmol electrons m^−2^ s^−1^ on 11, 12, and 20 December, respectively (Figure 3I). Therefore, it was evident that the cold snap largely decreased diurnal *J*_PSII_ values for this alpine species.

**Figure 4:**
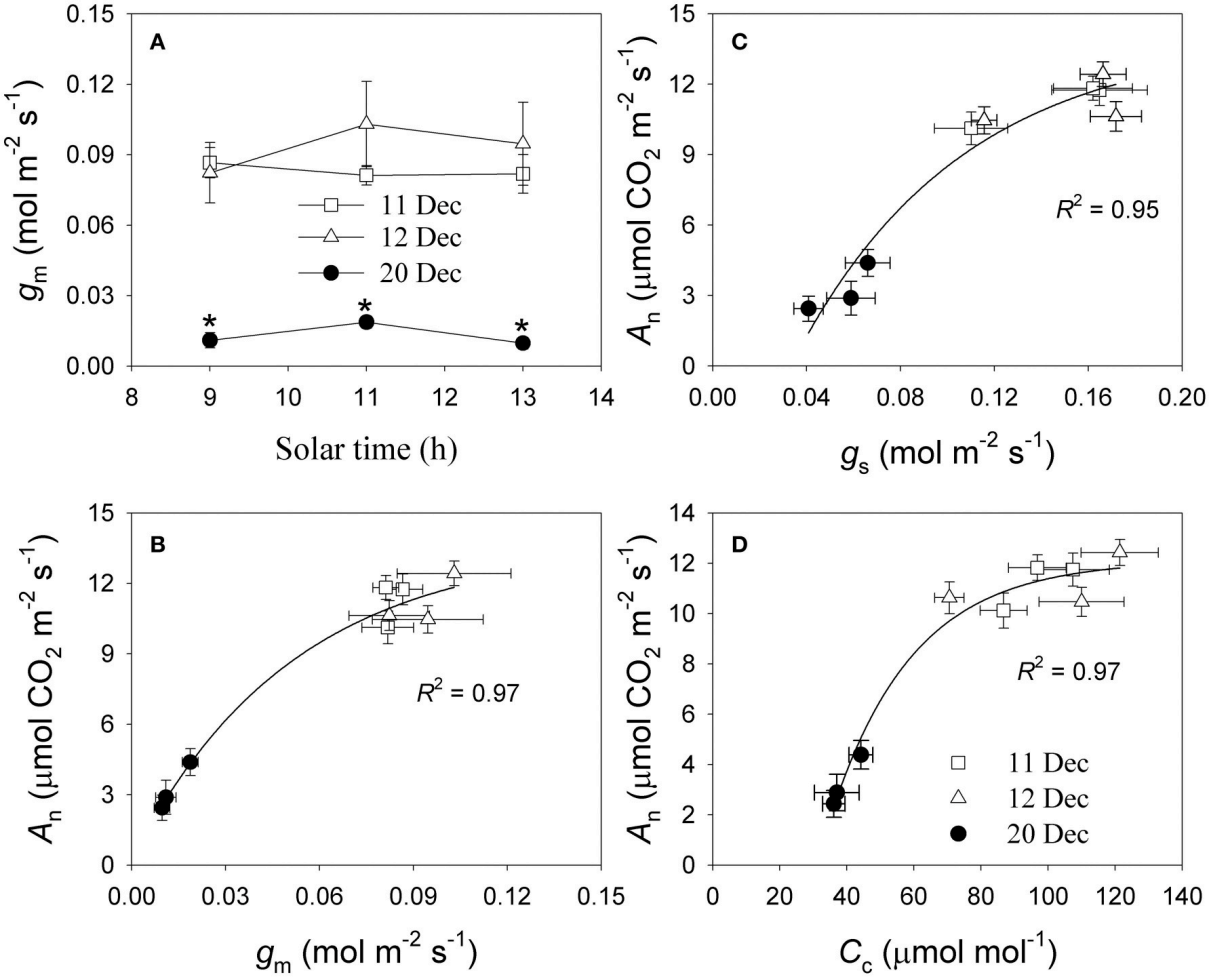
**(A)** Diurnal measurements of mesophyll conductance (*g*_m_); **(B)** correlation between *g*_m_ and net CO_2_ assimilation rate (*A*_n_); **(C)** correlation between stomatal conductance (*g*_s_) and photosynthetic rate (*A*_n_); and **(D)** correlation between chloroplast CO_2_ concentration (*C*_c_) and *A*_n_. Data for *g*_m_, *g*_s_, *C*_c_, and *A*_n_ were obtained at 9:00 h, 11:00 h and 13:00 h on 11, 12, and 20 December 2013. Asterisks indicate significant differences on 20 December when compared to 11 and 12 December.

To examine whether the depression in photosynthetic rates after the cold snap was caused by decreases in *g*_s_ and/or *g*_m_, we pooled the data for *g*_s_, *g*_m_, and *A*_n_ that were recorded on each date between 09:00 h and 13:00 h (when PPFD >1000 μmol photons m^−2^ s^−1^). Values for *g*_m_ at 09:00 h, 11:00 h, and 13:00 h were largely lower on 20 December (0.01 to 0.02 mol m^−2^ s^−1^) than on 11 and 12 December (range of 0.08 to 0.10 mol m^−2^ s^−1^) (Figure 4A). We also noted significant and positive relationships among *g*_s_, *g*_m_, and *A*_n_ for each of those dates (Figures 4B,C). Furthermore, the lower *g*_s_ and *g*_m_ on 20 December decreased values for *C*_c_, which directly constrained photosynthetic rate (Figure 4D). These results suggested that, after a cold snap, *g*_s_ and *g*_m_ were the two major determinants of *A*_n_ in winter for leaves of *Q. guyavifolia*.

**Figure 5:**
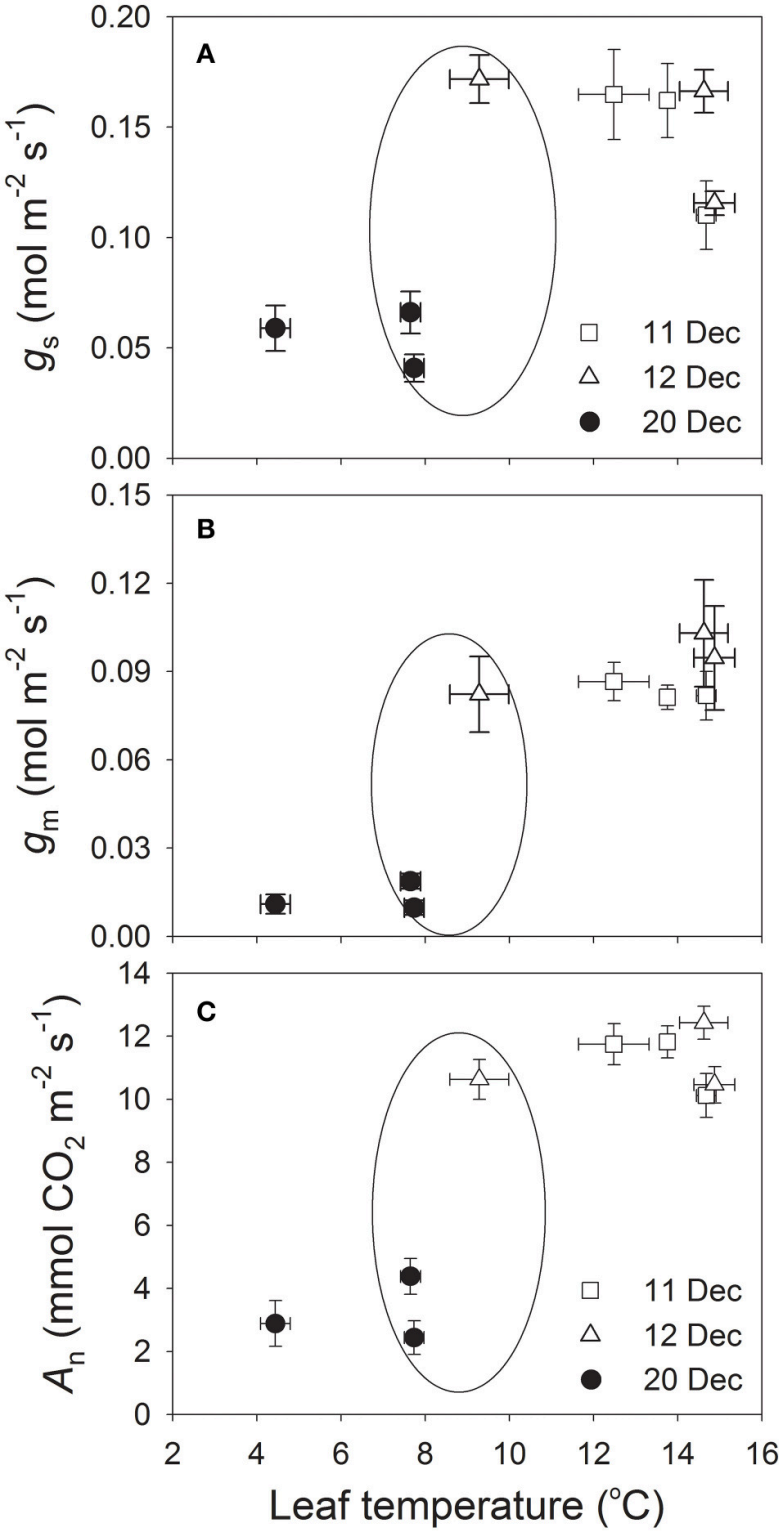
**Changes in stomatal conductance (*g*_s_) (A), mesophyll conductance (*g*_m_) (B), and photosynthetic rate (*A*_n_) (C) as a function of leaf temperature at 9:00 h, 11:00 h and 13:00 h on 11, 12, and 20 December 2013**.

**Figure 6:**
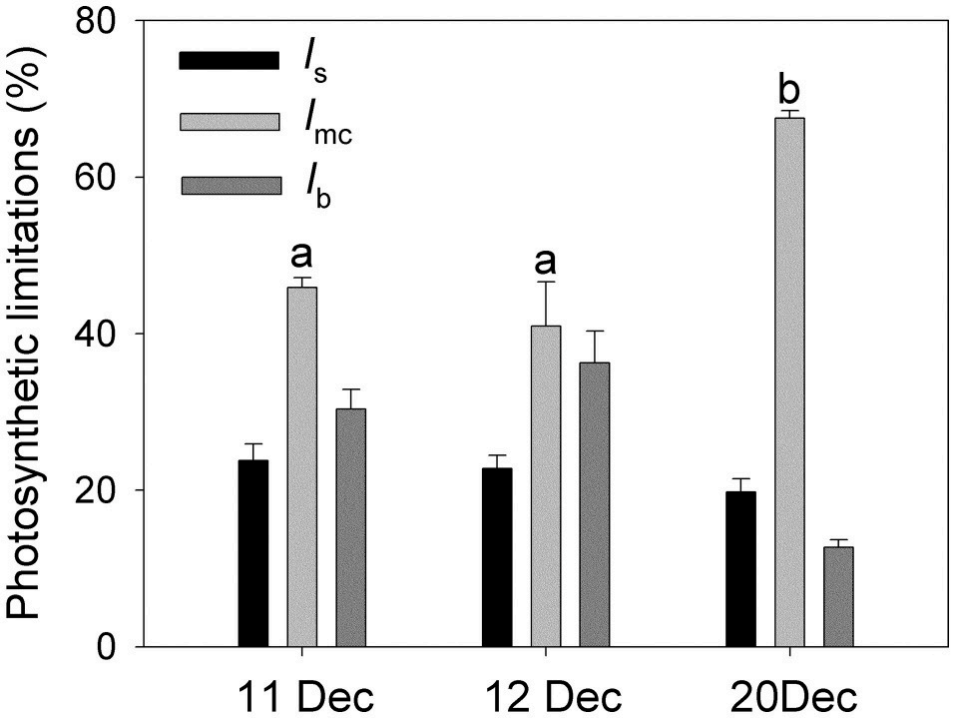
**Relative stomatal (*l*_s_), mesophyll conductance (*l*_mc_) and biochemical (*l*_b_) photosynthetic limitations in at 11:00 h on 11, 12, and 20 December 2013**. Different letters indicate significant differences among 11, 12, and 20 December.

To determine whether the decreases in *g*_s_ and *g*_m_ on 20 December were induced by the decrease in leaf temperature, the effect of leaf temperatures on *g*_s_, *g*_m_, and *A*_n_ were examined. Interestingly, similar leaf temperatures were accompanied with large differences in *g*_s_, *g*_m_, and *A*_n_ (Figures 5A–C). As a result, leaf temperature was not a major determinant of *g*_s_, *g*_m_, and *A*_n_ in winter for *Q. guyavifolia* leaves. The quantitative limitation analysis (Figure 6) revealed that, on 11 and 12 December the mesophyll conductance (*l*_mc_) was the largest (0.41–0.46), followed by biochemical (*l*_b_) and stomatal (*l*_s_) limitations. By comparison, on 20 December the mesophyll conductance was by far the most important (0.67), while stomatal (0.20) and biochemical (0.13) limitations were much less important. The mesophyll conductance limitation on 20 December was significantly higher than that on 11 and 12 December. These results suggested that the depression of *A*_n_ on 20 December was mainly caused by the decrease in *g*_m_ in response to lower night temperatures.

**Figure 7:**
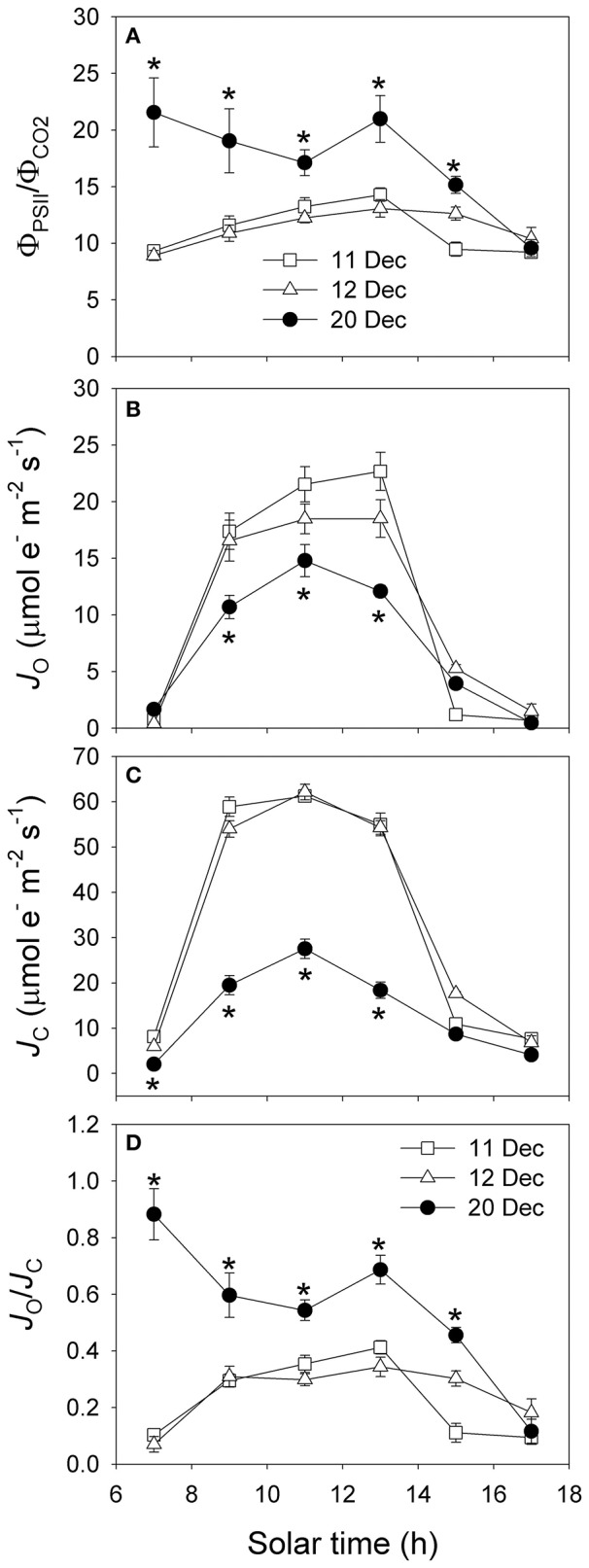
**Diurnal changes in Φ_PSII_/Φ_CO_2__ (A), *J*_O_ (B), *J*_C_ (C), and *J*_O_/*J*_C_ (D) on 11, 12, and 20 December 2013**. *J*_O_, electron flow devoted to oxygenation of RuBP. *J*_C_, electron flow devoted to carboxylation of RuBP. Values are means ± SE (*n* = 5). Asterisks indicate significant differences on 20 December when compared to 11 and 12 December.

**Figure 8:**
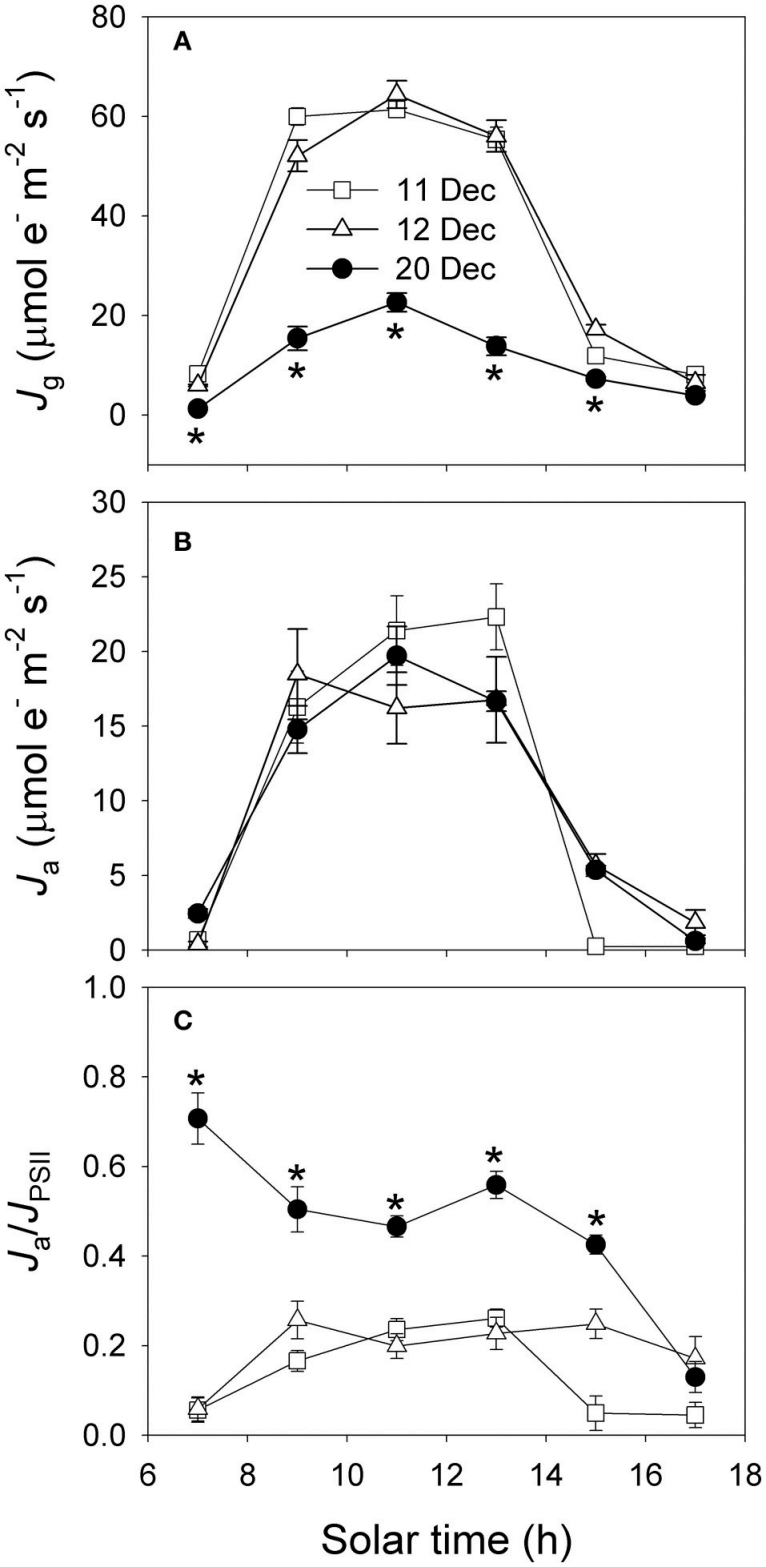
**Diurnal changes in *J*_g_ (A), *J*_a_ (B), and *J*_a_/*J*_PSII_ (C) on 11, 12, and 20 December 2013**. *J*_g_, electron flow devoted to production of NADPH calculated using data of gas exchange. *J*_a_, alternative electron flow calculated from the difference between *J*_PSII_ and *J*_g_. Values are means ± SE (*n* = 5). Asterisks indicate significant differences on 20 December when compared to 11 and 12 December.

The diurnal values of Φ_PSII_/Φ_C_O__2__ under high light were largely higher on 20 December than 11 and 12 December (Figure 7A). The rates of electron flow for photorespiration (*J*_O_) and RuBP carboxylation (*J*_C_) were lower on 20 December (Figures 7B,C). However, values for the *J*_O_/*J*_C_ ratio under high light were much higher on 20 December (Figure 7D), suggesting that photorespiration was the most important primary metabolism consuming ATP and NADPH when photosynthesis was restricted on 20 December. The rates of electron flow devoted to production of NADPH (*J*_g_) under high light on 20 December were much lower than that on 11 and 12 December (Figure 8A). However, the values of alternative electron flow (*J*_a_) under high light were similar on these 3 days (Figure 8B). As a result, the *J*_a_/*J*_g_ values under high light were largely higher on 20 December (Figure 8C). Therefore, alternative electron flow became a more important electron sink once photosynthesis was inhibited following decreased night temperature. Alternative electron flow generates proton gradient across thylakoid membrane (ΔpH) that can help production of extra ATP and activates non-photochemical quenching (NPQ). We found that the rates of ATP supplied from other flexible pathways (*v*_ATP(Flex)_) was much lower on 20 December. Furthermore, the same values of *J*_a_ was accompanied with much lower *v*_ATP(Flex)_ on 20 December. These results suggested that, on 11 and 12 December alternative electron flow mainly supplied ATP to drive both the Calvin cycle and photorespiration. By comparison, on 20 December alternative electron flow mainly induced strong lumen over-acidification, which in turn represses photosynthesis due to photosynthetic control and dissipation of excitation energy in the antenna bed.

## Discussion

Alpine evergreen broadleaf tree species typically experience cold temperatures at daytime/night during spring and winter. For plants grown at lower elevations, this type of stress would lead to strong declines in photosynthesis. However, it is unclear how such conditions affect photosynthesis in alpine evergreen broadleaf tree species. We found that the night air temperature was an important determinant of stomatal/mesophyll conductance and photosynthesis. When photosynthesis was inhibited following freezing night temperature, photorespiration and alternative electron flow were two important electron sinks to dissipate excess excitation energy.

### Stomatal/mesophyll conductances and photosynthetic rate in winter

Photosynthesis is one of the most temperature-sensitive processes in plants (Yamori et al., 2010). Although, the combination of chilling and intense light represents extreme growing conditions for many C_3_ plants, *Q. guyavifolia* appears to be well-adapted, as indicated by its high rate of photosynthesis on 11 and 12 December 2013. Light response curves indicated that the maximum rate of photosynthesis for this species in the summer time was 13 μmol CO_2_ m^−2^ s^−1^ (Figure 2). Therefore, our current results demonstrated that photosynthesis in *Q. guyavifolia* remains fully functional during the winter without cold snap. Previous investigations with other plant species have shown that night chilling (at approximately 5°C) can induce a significant decline in *g*_s_ and inhibit photosynthesis at midday in tomato, coffee, mango, grape, *Linociera insignis*, and *Calophyllum polyanthum* (Martin et al., 1981; Bauer et al., 1985; Flexas et al., 1999; Allen et al., 2002; Feng and Cao, 2005). In contrast, we found that exposure to 0°C low night temperatures was accompanied with high levels of *g*_s_ and *A*_n_ at midday in the alpine tree species *Q. guyavifolia*. Therefore, the responses of *g*_s_ and *A*_n_ to low night temperatures differed greatly between species.

Stomatal behavior is a complex process that can be regulated by VPD and temperature. A high VPD induces the stomata to close whereas a lower VPD triggers their opening (Lawson et al., 2014). On 11, 12, and 20 December, the maximum VPD was approximately 1.2 Kpa. Although, midday values for VPD were somewhat higher on 11 and 12 December than on20 December, *g*_s_ was significantly higher on the 11 and 12th. Therefore, these findings indicated that VPD is not an important factor for determining stomatal conductance in alpine evergreen tree species in winter. Pooling of the data for *g*_s_ and *A*_n_ collected between 9:00 and 13:00 h on our three test dates showed that wintertime photosynthetic rates are largely dependent upon *g*_s_. This suggested that stomatal conductance is a significant limiting factor for photosynthesis in *Q. guyavifolia* after a cold snap has occurred. This decrease in predawn water potential would also affect stomatal conductance and net photosynthesis. We found that, after a cold snap, the freezing night temperature significantly induced a leaf water deficit in our plants, where predawn leaf water potentials were < −2MPa (unpublished data). It seems that water deficit could be an additional factor for the decreases found in photosynthetic traits after a cold snap.

Mesophyll conductance can play an important role in determining photosynthetic rates (Carriquí et al., 2015), and can be affected by environmental conditions including leaf water status (Flexas et al., 2002; Galmes et al., 2007) and temperature (Bernacchi et al., 2002; Scafaro et al., 2011; Walker et al., 2013). Interestingly, values for *g*_m_ were significantly lower on 20 December than 11 and 12 December. Such a reduction in *g*_*m*_ can induce a drop in the chloroplast CO_2_ concentration (Flexas et al., 2002; Long and Bernacchi, 2003; Warren and Dreyer, 2006). Furthermore, a decrease in *g*_s_ can aggravate such a decline in *C*_c_ (Flexas et al., 2002). Our results indicated that diurnal values for *C*_c_ were much lower on 20 December than 11 or 12 December. The positive correlations among *g*_m_, *g*_s_, *C*_c_, and *A*_n_ indicated that lower levels of *g*_s_ and *g*_m_ on 20 December led to the decrease in *C*_c_, which then directly restricted the rate of CO_2_ assimilation. A quantitative limitation analysis revealed that, the mesophyll conductance limitation largely increased on 20 December compared to 11 and 12 December. Meanwhile, the biochemical limitation largely decreased on 20 December. This result suggested that the large decline in *A*_n_ on 20 December was independent of the activities of enzymes involved in photosynthesis, but mainly caused by the large decrease in *g*_m_.

In alpine zones, cold snaps largely decrease air and leaf temperatures. Likewise, the leaf temperature was 9.3°C and *A*_n_ was 11.7 μmol m^−2^ s^−1^ at 09:00 h on 12 December vs. 7.6°C and 4.4 μmol m^−2^ s^−1^, respectively, at 11:00 h on 20 December. The large gap in *A*_n_ between those two dates was associated with only a slight change in the leaf temperature, thereby implying that leaf temperature is not a major limiting factor for CO_2_ assimilation. Similarly, values for *g*_s_ and *g*_m_ were significantly higher at 09:00 h on 12 December than at 11:00 h on 20 December. This again indicated that the declines in *g*_s_ and *g*_m_ after the cold snap were not primarily caused by a change in the leaf temperature. Apparently, the diurnal *g*_s_, *g*_m_, and *A*_n_ were significantly depressed when the minimum night air temperature dropped to −7°C on 20 December. Freezing conditions overnight can induce icing in the xylem and leaves, which then reduces stem and leaf hydraulic conductivity and elicits a leaf water deficit that suppresses *g*_s_ and *g*_m_. Furthermore, the chilling of roots can depress photosynthesis due to a decrease in *g*_s_ (Day et al., 1991; Bassirirad et al., 1993). Nevertheless, the soil temperature changed slightly before and after the cold snap (decreasing about 1°C, data not shown). Therefore, we assume that freezing night air temperature markedly affected daytime values of *g*_s_ and *g*_m_, and the night air temperature would be a critical environmental factor determining diurnal values of *g*_s_, *g*_m_, and *A*_n_ in winter.

### Regulation of photosynthetic electron flow in winter

The rates of both CO_2_ assimilation and photosynthetic electron flow under high light on 20 December were significantly lower than that on 11 and 12 December (Figures 3C,H), but the ratio of Φ_PSII_ to Φ_C_O__2__ was significantly higher on 20 December (Figure 7A). This increase in Φ_PSII_/Φ_C_O__2__ demonstrated that more of the electron transfer from PSII to PSI was being consumed through the photorespiratory pathway and/or alternative electron sinks. The affinity of Rubisco is mainly affected by temperature and intercellular CO_2_ concentrations (von Caemmerer, 2000). On 20 December, the decrease in *C*_c_ increased the affinity of Rubisco to O_2_ and then aggravated photorespiration, as indicated by the increased values of *J*_O_/*J*_C_ (Figure 7D). Photorespiration consumes excess NADPH and, thus, alleviates its over-accumulation on the acceptor side of PSI, preventing the over-reduction of photosynthetic electron chains (Huang et al., 2015b). Thus, photorespiration plays a significant role in consuming excess excitation energy in the alpine evergreen tree species *Q. guyavifolia*. In sun leaves of *Ranunculus glacialis*, up to 40% of the photosynthetic electron flow is consumed through alternative electron sink (Laureau et al., 2013). In *Q. guyavifolia*, values for *J*_a_/*J*_PSII_ ratio significantly increased on 20 December and reached to 50% under high light. As a result, alternative electron flow was a major electron sink in *Q. guyavifolia* when photosynthesis was inhibited in winter. The PTOX protein, which is critical to that alternative sink in alpine plants, can transfer electrons from plastoquinone to molecular oxygen. In fact, all measured alpine plant species contain high amounts of PTOX protein (Streb et al., 2005; Laureau et al., 2011, 2013). Therefore, it is conceivable that *Q. guyavifolia* also has a high PTOX content that can induce greater alternative electron flow when CO_2_ assimilation is restricted after cold snaps. Moreover, PTOX can keep the plastoquinol pool oxidized under cold, heat, or high-light stresses, and thus alleviate photoinhibition of PSII.

**Figure 9:**
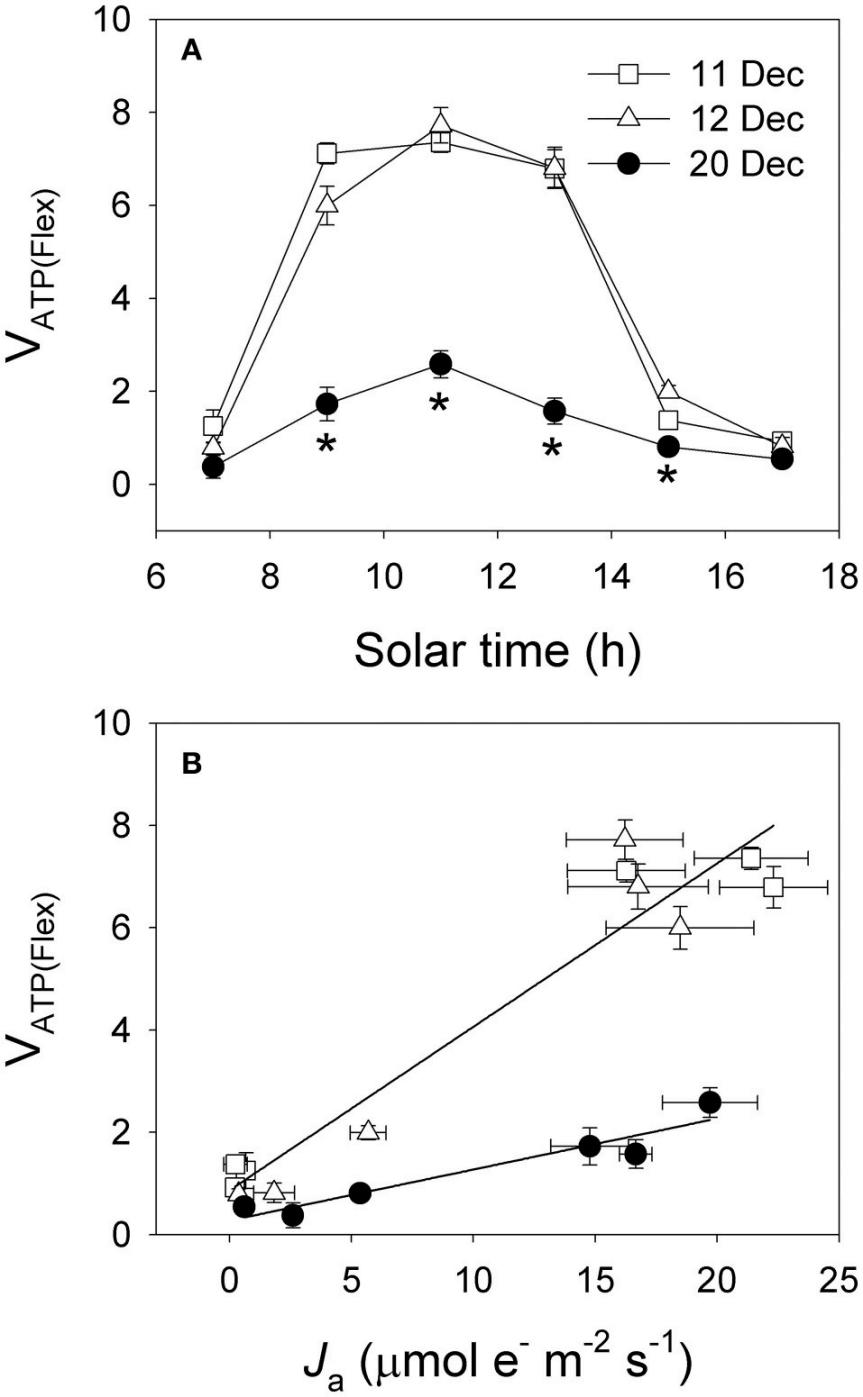
**(A)** Diurnal changes in the rates of ATP supplied from other flexible pathways (*v*_ATP(Flex)_). **(B)** Relationship between the WWC activity (*J*_a_) and *v*_ATP(Flex)_. Values are means ± SE (*n* = 5). Asterisks indicate significant differences on 20 December when compared to 11 and 12 December.

Alternative electron flow assists in the generation of a proton gradient across the thylakoid membranes (ΔpH), which helps extra ATP production and/or accelerates lumen acidification (Miyake, 2010). Under conditions of high rates of photosynthesis, the main role of alternative electron flow was to supply ATP for the primary metabolism including the Calvin cycle, photorespiration and nitrogen assimilation (Makino et al., 2002; Huang et al., 2016). However, under conditions of high light and low rate of photosynthesis, alternative electron flow mainly contributes to lumen acidification. On 11 and 12 December, the high levels of photosynthetic CO_2_ assimilation and photorespiration needed more ATP supplied from other flexible mechanisms (*v*_ATP(Flex)_) (Figure 9A). We found that, under high light the same values of *J*_a_ were accompanied with much higher values of *v*_ATP(Flex)_ on 11 and 12 December when compared to 20 December (Figure 9B). This result strongly suggested that alternative electron flow mainly functioned for ATP synthesis on 11 and 12 December, but primarily induced lumen acidification on 20 December. An increase in lumen acidification not only activates the photoprotective NPQ mechanism but also controls linear electron transfer from PSII to PSI via Cyt *b*_**6**_/*f* (Tikkanen and Aro, 2014; Chaux et al., 2015; Suorsa, 2015; Tikkanen et al., 2015). Consistently, the diurnal values for *F*_*v*_′/*F*_*m*_′ on 20 December were significantly lower than that on 11 and 12 December. Because *F*_*v*_′/*F*_*m*_′ is inversely related to NPQ, this result provides evidence for stronger activation of NPQ on 20 December. When CO_2_ assimilation under intense irradiance is depressed by freezing night temperatures, as shown here, the alternative electron flow in *Q. guyavifolia*l leads to rapid over-acidification of thylakoid lumen and then protects the photosynthetic apparatus. Therefore, alternative electron flow may be an important strategy used by the alpine evergreen tree species *Q. guyavifolia* to regulate photosynthetic electron flow and diminish the risk of photodamage in winter. We noted here that the maximum quantum yield of PSII (*F*_*v*_/*F*_*m*_) and maximum photo-oxidizable P700 (*P*_*m*_) in winter were maintained stable compared to summer (data not shown), indicating the stability of PSI and PSII activities in winter.

## Conclusion

In the present study, we found that night chilling temperature had little effect on daytime values of stomatal/mesophyll conductance and photosynthesis in *Q. guyavifolia*. However, when the minimum air temperature at night decreased to −7°C, stomatal and mesophyll conductance were largely reduced in the daytime, which led to lower chloroplast CO_2_ concentrations and the inhibition of photosynthesis. Similar leaf temperature was accompanied with large differences in stomatal/mesophyll conductance and photosynthesis on 3 days with different night air temperature. Therefore, night air temperature played a significant role in determining stomatal/mesophyll conductance and photosynthesis in the alpine evergreen tree species *Q. guyavifolia*. When photosynthesis was inhibited following freezing night temperature, photorespiration and alternative electron flow played important roles in regulation of photosynthetic electron flow and protected the photosynthetic apparatus against photodamage.

## Author contributions

Conceived and designed the experiments: WH, SZ, and HH; Performed the experiments: WH and SZ; Analyzed the data: WH and SZ; Contributed reagents/materials/analysis tools: WH, SZ, and HH; Contributed to the writing of the manuscript: WH, SZ, and HH.

## Funding

This work was supported by the National Natural Science Foundation of China (Grants 31300332 and 31670343), the foundation from Chinese Academy of Science (QYZDY-SSW-SMC014) and Yunnan Applied Basic Research Project (Grant 2016FB054).

### Conflict of interest statement

The authors declare that the research was conducted in the absence of any commercial or financial relationships that could be construed as a potential conflict of interest.
